# Physicochemical Characterization, Skin Penetration, Anti-Melanogenesis and Safety Assessment of Flavokawain C Nanofibers

**DOI:** 10.3390/ijms26072966

**Published:** 2025-03-25

**Authors:** Pamela Berilyn So, Ying-Chu Wang, Pao-Hsien Huang, Tzu-Hui Wu, Feng-Lin Yen

**Affiliations:** 1 Department of Fragrance and Cosmetic Science, College of Pharmacy, Kaohsiung Medical University, Kaohsiung 807378, Taiwan; pam@kmu.edu.tw; 2 Department of Medical Research, Kaohsiung Medical University Hospital, Kaohsiung 807378, Taiwan; 3 Doctoral Degree Program in Toxicology, College of Pharmacy, Kaohsiung Medical University, Kaohsiung 807378, Taiwan; u107012028@gap.kmu.edu.tw; 4 School of Pharmacy, College of Pharmacy, Kaohsiung Medical University, Kaohsiung 807378, Taiwan; j6466497@gmail.com; 5 Department of Pharmacy and Master Program, Collage of Pharmacy and Health Care, Tajen University, Pingtung 90741, Taiwan; 6 Institute of Biomedical Sciences, National Sun Yat-Sen University, Kaohsiung 80424, Taiwan; 7 Drug Development and Value Creation Research Center, Kaohsiung Medical University, Kaohsiung 80756, Taiwan; 8 College of Professional Studies, National Pingtung University of Science and Technology, Pingtung 912301, Taiwan

**Keywords:** flavokawain C, nanofibers, skin whitening, cosmetic safety assessment

## Abstract

Various whitening cosmetics are available in the market, usually containing active whitening ingredients. However, most of the reported active ingredients have low dermal penetration due to their lipophilic structure. Therefore, it is necessary to develop effective whitening agents and novel formulations to address this. In previous studies, natural compounds such as chalcones have shown inhibitory effects on tyrosinase. However, most chalcone compounds have the disadvantage of poor water solubility, which restricts their dermal absorption. Flavokawain C (FKC) is a natural chalcone obtained from the root of the kava tree (*Piper methysticum*) and can also be obtained through organic synthesis. Since FKC is a chalcone, it is also water-insoluble, showing poor dermal absorption. In this study, electrospinning technology was used to develop FKC nanofibers (FKCNFs) to improve FKC’s physicochemical properties. The results showed that FKCNFs significantly improved water solubility and percutaneous absorption. Based on the results of in vitro experiments with B16F10 melanoma cells, 10 µM FKCNFs repressed the expressions of melanogenesis-related proteins MITF and TRP2. Furthermore, cosmetic safety assessment revealed that FKCNFs displayed a good margin of safety. This study suggests that FKCNFs have great potential as an effective active ingredient for whitening cosmetics.

## 1. Introduction

Under normal physiological conditions, melanin deposition protects human skin from UV damage. However, excessive synthesis of melanin in the skin can lead to many skin diseases, such as chloasma, sun spots, freckles, and age spots, which cause uneven skin color. Melanogenesis is a complex series of enzymatic and chemical reactions. When exposed to UV rays, paracrine cytokines including α-melanocyte-stimulating hormone (α-MSH), stem cell factor (SCF), and nitric oxide (NO) promote melanin production [1]. When α-MSH binds to melanocortin 1 receptor (MC1R), it activates the cyclic adenosine monophosphate/protein kinase A (cAMP/PKA) pathway, stimulates the phosphorylation of cAMP-response element-binding protein (CREB), affects the transcription of microphthalmia-associated transcription factor (MITF), and promotes the production of melanin-related proteins, such as tyrosinase, tyrosinase-related protein-1 (TRP1), and tyrosinase-related protein-2 (TRP2) [2]. These proteins catalyze the production of melanin. In addition, SCF/c-kit signaling is closely related to the development of melanocytes [3]. When SCF binds to the c-kit receptor on the cell surface, it activates mitogen-activated protein kinases (MAPKs). MAPK family proteins include ERK, JNK, and p38. Previous literature has reported that the phosphorylation of MAPK family proteins can phosphorylate MITF, leading to its degradation and reducing the synthesis of melanin by melanocytes [4,5,6]. Furthermore, p38 phosphorylation promotes MITF activation and upregulates the expression of melanin production-related proteins, ultimately causing melanin production [1,7,8]. In the same way, tyrosinase is necessary for the production of melanin. There are many whitening products that inhibit the production of melanin by inhibiting tyrosinase. However, the tyrosinase inhibitors that are currently used all possess some disadvantages, such as kojic acid, which is slightly photo-irritant [9] and is unstable during storage [10]. Arbutin is easily degraded into hydroquinone at high temperatures, which may potentially cause bone marrow toxicity [11]. Therefore, it is necessary to develop new tyrosinase inhibitors to reduce the risk of adverse side effects.

Chalcones are natural products that are widely distributed in fruits, vegetables, spices, tea, and soy foods. Previous research has shown that chalcones possess an inhibitory effect on tyrosinase [12,13]. Kava (*Piper methysticum*) is native to the tropical island regions in the Pacific. It has been used for hundreds of years in religious rituals and its extracts have also been used as a dietary supplement to treat stress, anxiety, insomnia, restlessness, and muscle pain [14,15,16]. One of the active naturally occurring chalcones extracted from kava is Flavokawain C (FKC). Studies show that FKC possesses good anticancer activity [17,18,19], and also anti-melanogenesis activity [20]. Related literature shows that FKC has a better IC_50_ value and better anti-melanin activity as compared to kojic acid [20]. Chalcones are α,β-unsaturated carbonyl compounds that can be used for skin whitening [13,21], anti-inflammation [22,23], and anticancer properties [17,24,25]. However, because its chemical structure contains benzene rings and carbonyl structures, the water solubility of the compound is low. Several techniques to enhance its water solubility include using sodium saccharin or sodium cyclamate for co-solubilization [26], and development as a sodium phosphate prodrug [27]. Another promising approach to improve water solubility is through the use of electrospinning technology, incorporating the active ingredients into nanofibers, to improve the problem of lipophilic structures that are not easily soluble in water and affect their percutaneous absorption rate. Electrospinning is a technique used for preparing nanofibers, wherein their diameter can be reduced from hundreds of microns to tens of nanometers [28]. Nanofibers made using electrospinning technology have the characteristics of high porosity, high surface area, and high moisture permeability. They are widely used in the biomedical field, such as wound dressings, tissue engineering scaffolds, and transdermal drug delivery systems [29], allowing efficient dermal absorption of actives. Materials used for electrospinning technology are usually polymers, due to their flexibility and processability [30]. 2-hydroxypropyl-β-cyclodextrin (HPBCD) and polyvinylpyrrolidone (PVP) are Generally Recognized As Safe (GRAS) excipients. These pharmaceutical-grade polymers were selected based on their established regulatory compliance and demonstrated efficacy in enhancing the bioavailability of hydrophobic active compounds through solubility modification and molecular encapsulation mechanisms. In our previous study, we successfully encapsulated ursolic acid and improved its water solubility by the preparation of ursolic acid nanofibers (UANFs) [31].

This study is mainly divided into three parts: nanofiber preparation and characterization, in vitro efficacy test, and safety assessment. After FKCNF preparation, the physical and chemical properties will be assessed. The best FKCNF ratio will be selected and used for subsequent efficacy tests, including cell viability assay, melanogenesis inhibition using B16F10 cells, and determination of the underlying mechanism of the inhibition using Western blot. Finally, a cosmetic safety assessment will be conducted, wherein the margin of safety (MoS) of cosmetic raw materials will be calculated through percutaneous absorption experiments.

## 2. Results and Discussion

### 2.1. Material Characterization

**Figure 1:**
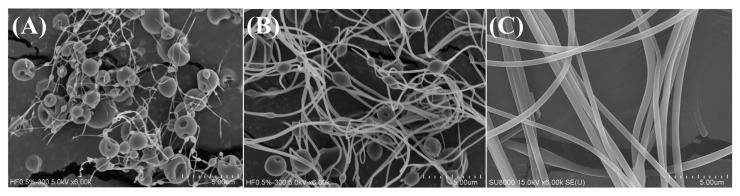
Material characterization. Surface morphology of FKCNF with different ratios (FKC:HPBCD:PVP, *w*/*w*/*w*) observed using SEM. (**A**) FKCNF (1:50:5), (**B**) FKCNF (1:50:10), and (**C**) FKCNF (1:50:20).

The preparation of FKCNFs using HPBCD, PVP, and FKC via electrospinning proved to be successful, with the SEM images in Figure 1C below showing the fibrous structures of FKCNFs (1:50:20). The morphologies of HPBCD, PVP, and FKC as raw materials prior to electrospinning are also shown in Figure S1 for comparison. HPBCD has a hollow spherical shape, PVP is seen as irregularly shaped flakes, and FKC shows irregular, non-uniform block-like particles. It can also be observed that FKCNF ratios of 1:50:5 (Figure 1A) and 1:50:10 (Figure 1B) showed granular shapes with thin nanofibers, while an FKCNF ratio of 1:50:20 showed a uniform fibrous morphology, with an average diameter of 513.60 ± 77.25 nm. It can be observed that the amount of PVP in the formulation is directly proportional to the nanofiber diameter. Attempts were made to prepare nanofibers with a ratio of 1:50:40 (FKC:HPBCD:PVP). However, the high proportion of PVP increased the viscosity of the solution, which in turn made it impossible to produce nanofibers during the electrospinning process.

**Figure 2:**
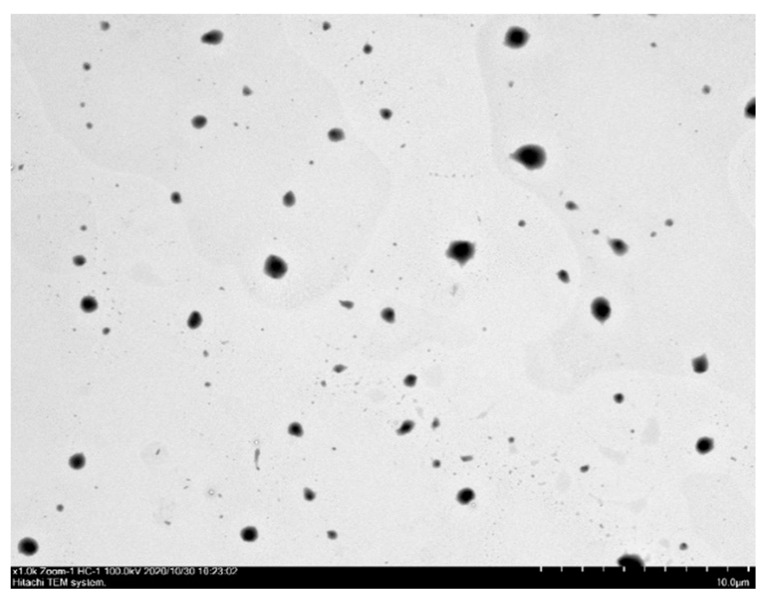
FKCNFs in aqueous solution observed under TEM.

The particle size of FKCNFs at a concentration of 10 μg/mL was approximately 590.90 ± 54.52 nm, with a polydispersity index (PDI) of 0.37 ± 0.03, showing a uniform particle size distribution. Moreover, observation using TEM, shown in Figure 2, showed that the average particle size was 478.69 ± 222.27 nm, which corroborates the data above showing that the FKCNFs are approximately 500 nm in size.

**Figure 3:**
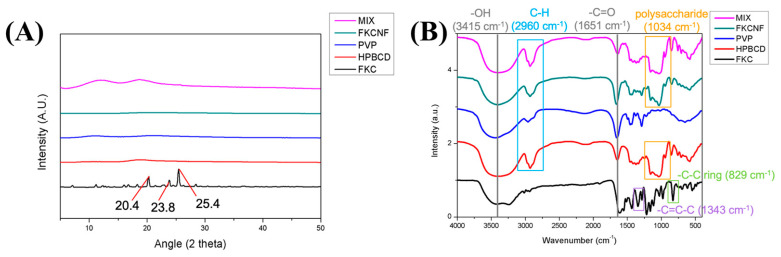
Comparative structural characterization of FKC, excipients, and FKCNF: (**A**) powder X-ray diffraction (PXRD) patterns and (**B**) Fourier transform infrared (FTIR) spectra of Flavokawain C (FKC), 2-hydroxypropyl-beta-cyclodextrin (HPBCD), polyvinylpyrrolidone (PVP), Flavokawain C nanofibers (FKCNFs) (1:50:20), and physically mixed (HPBCD:PVP:FKC, 1:50:20) powder (MIX).

The crystalline structural changes were detected using powder X-ray diffraction. As shown in Figure 3A, the characteristic diffraction peaks of FKC appear at 20.3°, 23.8°, and 25.4° 2 theta, indicating that FKC is in a crystalline state. All other components were amorphous in nature. For comparison, HPBCD, PVP, and FKCNF were physically mixed in a similar ratio (1:50:20), denoted as MIX, wherein the characteristic peaks of FKC were not clearly observed, due to the relatively small proportion of FKC in the formula. In the same way, FKCNFs showed no characteristic peak, indicating that the FKCNFs prepared by electrospinning were amorphous, thereby improving the water solubility of FKC. The FTIR spectra of the samples are shown in Figure 3B. FKC shows distinct absorption bands at 1651 cm^−1^ (for the -C=O functional group), 1343 cm^−1^ (for -C=C-C), and 829 cm^−1^ (for the -C-C ring). When FKC was combined with HPBCD and PVP to form FKCNFs, the intensity of the absorption bands between 1200 and 1500 weakened. Moreover, new peaks can be observed at 2960 cm^−1^ (for C-H) and 1034 cm^−1^ (for polysaccharides), showing that FKC forms intermolecular hydrogen bonds with the excipients. Similarly to previous reports, after the compound was made into nanofibers with HPBCD/PVP, the drug changed from a crystalline structure to an amorphous state, and new intermolecular bonds were formed [32,33]. These results indicate that FKC has been successfully incorporated into the nanofibers, improving the physicochemical properties of the original compound and thus enhancing its water solubility.

Because the solubility of FKC is below the minimum value (0.01 μg/mL) that the HPLC can detect, the water solubility of raw FKC was estimated to be less than 0.01 μg/mL. The calibration curve prepared for the 0.01–100 μg/mL concentration range showed a good linear relationship, which was used as the basis for the quantitative analysis of the subsequent FKCNF water solubility, yield, photostability, thermal stability, and transdermal absorption experimental results (Figure S2). The percent yield and solubility of the prepared FKCNFs are presented in Table 1. The results show that the preparation of FKC into nanofibers by electrospinning improves the water solubility of FKC by at least 40,000 times. Higher PVP ratios in the system lead to better solubility of FKC despite lower yields. When PVP is too low, the solution does not form fibers properly, leading to beads or irregular structures. This reduces surface area, so FKC is not as soluble. FKC partitioning into the PVP phase can keep FKC within the fibers, improving solubility. As the proportion of PVP increases, the water solubility of nanofibers also increases, wherein FKCNF (1:50:20) has the best water solubility of 515.32 μg/mL.

### 2.2. Stability Tests

**Figure 4:**
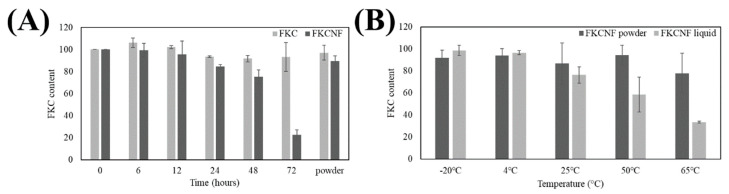
Stability tests for FKCNF. (**A**) Photostability upon exposure to artificial UV light and (**B**) thermal stability upon exposure to various temperatures. The FKC content of the samples was quantified using HPLC-UV. Data are expressed as mean ± SD (*n* = 3).

For photostability testing, the samples were exposed to artificial UV light irradiation, where the UVA energy is 0.96 J/cm^2^/h and the UVB energy is 1.9 J/cm^2^/h, at the following time intervals: 6 h (UVA energy of 5.76 J/cm^2^, UVB energy of 11.40 J/cm^2^), 12 h (UVA energy of 11.52 J/cm^2^, UVB energy of 22.80 J/cm^2^), 24 h (UVA energy of 23.04 J/cm^2^, UVB energy of 45.60 J/cm^2^), 48 h (UVA energy of 46.08 J/cm^2^, UVB energy of 91.20 J/cm^2^) and 72 h (UVA energy of 69.12 J/cm^2^, UVB energy of 136.8 J/cm^2^). The control group was not treated with light, and the powder group was dissolved into liquid after 72 h of light irradiation (UVA energy of 69.12 J/cm^2^, UVB energy of 136.8 J/cm^2^). It can be seen in Figure 4A that after 24 h, the FKC content decreased by 15.47%; after 48 h, it decreased by 24.72%; and after 72 h, it decreased by 77.4%. As the irradiation time increased, FKCNF showed a time-dependent decrease; in comparison, the content of FKC decreased by only 6.67% after 72 h of illumination, and the content of FKCNF in the powder group decreased by 10.69% after 72 h of illumination. In addition, the thermal stability of the FKCNF powder group is better than that of the solubilized group (FKCNF liquid form). As shown in Figure 4B, the FKC content drops to 76.45% at 25 °C, 58.56%, at 50 °C, and 33.56% at 65 °C. The results indicate that the thermal stability of the FKCNF solution group is poor at room temperature and in high-temperature environments, but it is more stable in the powder state. The results show that FKCNFs are more suitable for storage in a powder state than in solution form.

### 2.3. Cell Viability

**Figure 5:**
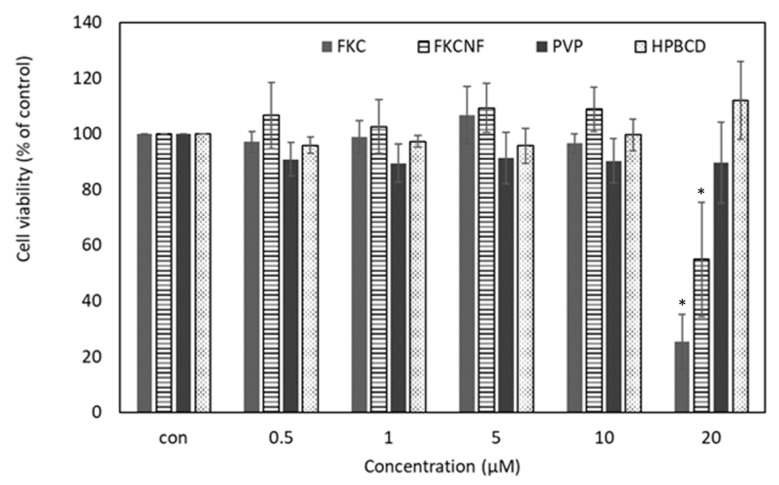
Effect of FKC, FKCNFs, PVP, and HPBCD on the cell viability of B16F10 mouse melanoma cells. Cytotoxicity was evaluated using the MTT assay. Data are expressed as mean ± SD (*n* = 3). * indicates a significant difference compared with the control group (*p* < 0.05).

The MTT assay was used to evaluate the cytotoxicity of FKC, FKCNF, HPBCD, and PVP in B16F10 mouse melanoma cells. Figure 5 shows that FKC and FKCNFs had obvious cytotoxicity at a concentration of 20 μM, reducing the cell survival rate to 25.35% and 54.87%, respectively. Therefore, subsequent cell experiments were conducted using only 5 and 10 μM FKC and FKCNFs.

### 2.4. Melanogenesis Inhibition

**Figure 6:**
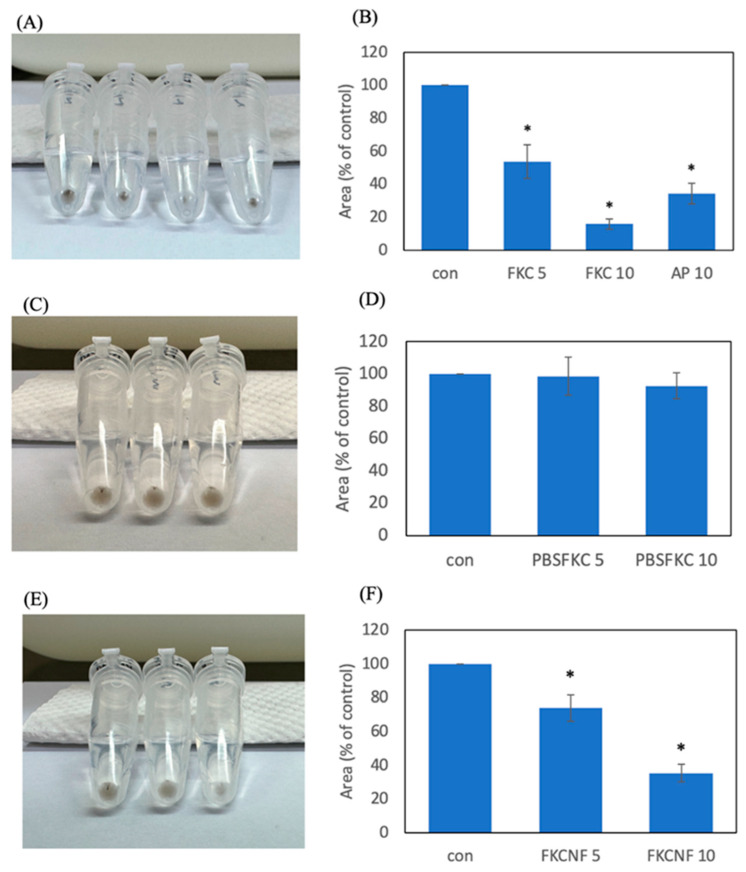
Effect of FKC and FKCNFs on the melanin content in B16F10 cells: (**A**,**B**) 1% DMSO-FKC group, (**C**,**D**) Raw-FKC group, and (**E**,**F**) FKCNF group, imaged and analyzed using Image J software v1.54g. Data are expressed as mean ± SD (*n* = 3). * indicates a significant difference compared with the control group (*p* < 0.05).

To investigate the effect of FKC and FKCNF on melanogenesis inhibition, the melanin concentration of B16F10 cells exposed to various concentrations of FKC, FKCNFs, and AP was measured. DMSO (1%) was used to solubilize FKC and ascorbyl palmitate (positive control), denoted as the % DMSO-FKC group. As shown in Figure 6A,B, using 1% DMSO to solubilize 5 and 10 μM FKC effectively decreased the melanin content to 53.79% and 15.97%, respectively, while the positive control, AP, was able to decrease melanin content to 34.34%. Without using DMSO, it can be observed that 5 and 10 μM FKC (denoted as the Raw-FKC group) cause no significant reduction in melanin content (Figure 6C,D). Interestingly, a significant reduction in melanin content can be seen in 5 and 10 μM FKCNFs (denoted as the FKCNF group) (Figure 6E,F), showing effective solubilization when FKC is prepared as nanofibers.

### 2.5. Expression of Melanogenesis-Related Proteins in B16F10 Cells

**Figure 7:**
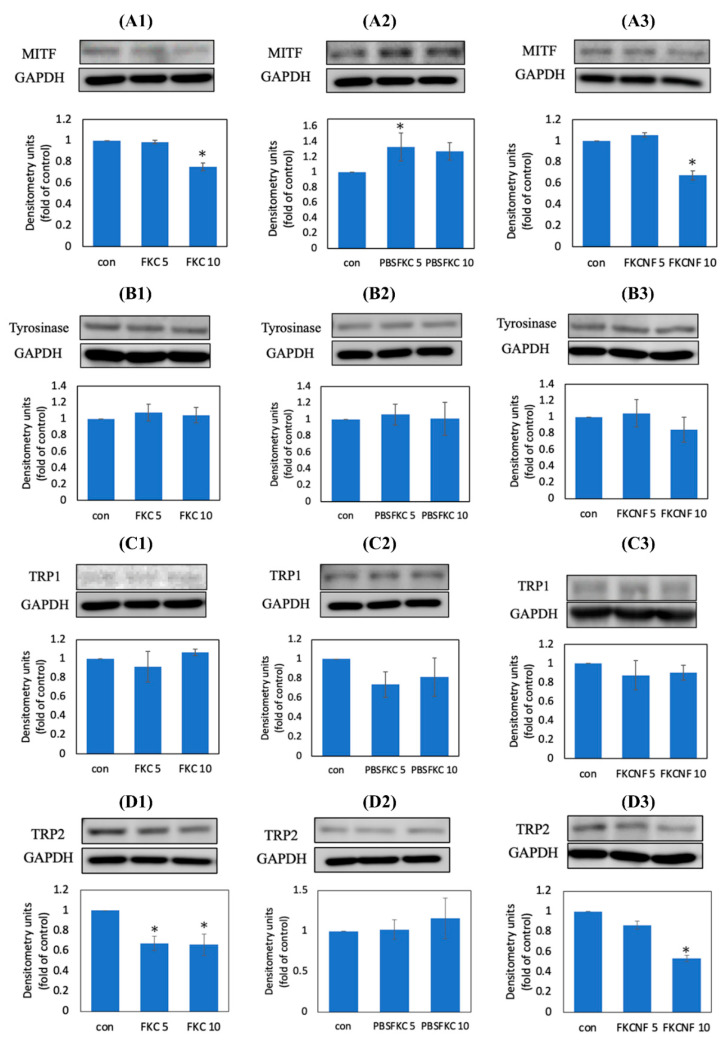
Effect of FKC on the expression of melanogenesis-related proteins in B16F10 cells via Western blot. Immunoblots and densitometric analysis of (**A**) MITF, (**B**) tyrosinase, (**C**) TRP1, and (**D**) TRP2. The numbers designate the sample groups: (**1**) 1% DMSO-FKC group, (**2**) Raw-FKC group, and (**3**) FKCNF group. GAPDH was used as an internal control. Data are expressed as mean ± SD (*n* = 3). * indicates a significant difference compared with the control group (*p* < 0.05).

Enzymes related to melanin production include tyrosinase, TRP1, and TRP2, which are the key enzymes that regulate melanin synthesis [12], and these three enzymes are mainly regulated by MITF. To test the effect of FKC on melanogenesis-related proteins, their effects on the expression of microphthalmia transcription factor (MITF), tyrosinase, tyrosinase-related protein-1 (TRP1), and tyrosinase-related protein-2 (TRP2) were investigated. Figure 7(A1) shows that 10 μM FKC can effectively inhibit the expression of the MITF protein by 24.51%, while in Figure 7(A2), 5 μM of raw FKC can increase the expression of MITF by 33.02%. Additionally, it can be observed in Figure 7(A3) that 10 μM FKCNF effectively reduces the expression of MITF by 33.39%. Meanwhile, Figure 7B,C show that FKC has no significant inhibitory effect on the expression of tyrosinase and TRP1, while in Figure 7D, it can be seen that 5 and 10 μM FKC in 1% DMSO, and 10 μM FKCNF can effectively inhibit the expression of TRP2 by 32.64%, 33.9%, and 46.6%, respectively. However, there are no significant changes in protein expression for the Raw-FKC group, which indicates that dispersing FKC in PBS cannot significantly inhibit the expression of melanogenesis-related proteins. The inability to exhibit biological activity is speculated to be due to the low water solubility of FKC, further proving the necessity of preparing FKC as a soluble preparation like FKCNFs. These results show that FKCNFs inhibit melanin production by inhibiting the expression of MITF and TRP2.

The MAPK pathway is one of the key regulators of melanogenesis. Phosphorylation of ERK, JNK, and p38 will affect the expression of MITF [1,7,34]. Therefore, the effect of FKC on the protein expression of ERK, JNK, and p38 was investigated. As shown in Figure S3A, 5 μM FKC in DMSO significantly increased p-ERK expression by 12.89 times. In the Raw-FKC group, 5 and 10 μM FKC significantly increased p-ERK expression by 18.05 and 19.38 times, respectively. In addition, 5 μM FKCNF significantly increased p-ERK expression by 16.64 times. In the same way, 10 μM FKC in DMSO significantly increased p-JNK expression by 3.65 times while FKCNFs led to an increasing trend in p-JNK expression (Figure S3B). Furthermore, 10 μM FKC significantly increased p-p38 expression by 1.86-fold, and 10 μM FKCNFs significantly increased p-p38 expression by 2.2-fold (Figure S3C). Based on these results, we infer that FKC and FKCNF upregulate MAPK phosphorylation, thereby achieving melanogenesis inhibition, confirming that FKC and its nanofibers have the potential to be developed as melanogenesis inhibitors. Original unprocessed images of blots are included in the Supplementary Information (Figures S4–S10).

### 2.6. Percutaneous Absorption and Margin of Safety (MoS)

**Figure 8:**
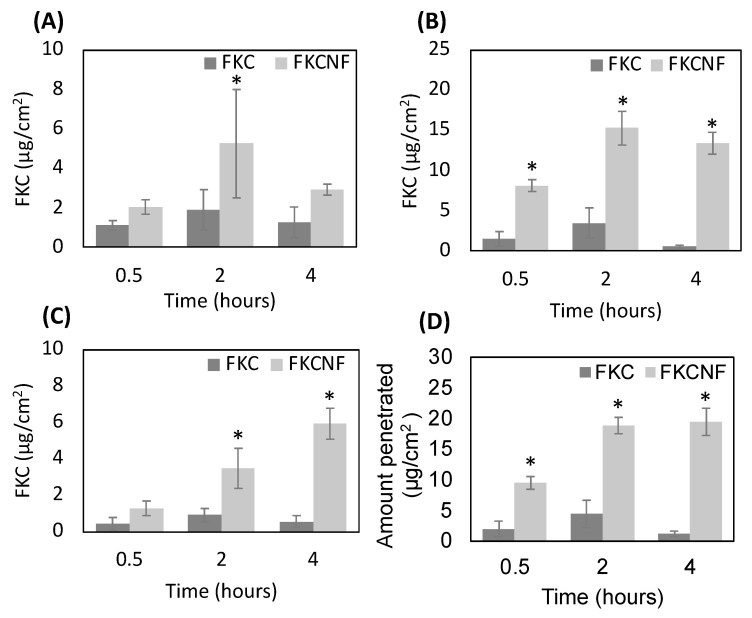
FKC content in different layers of pig skin after percutaneous absorption of FKC and FKCNF solutions in Franz diffusion cells: (**A**) stratum corneum, (**B**) epidermis, and (**C**) dermis. (**D**) Percentage of FKC content in the epidermis and dermis at different time points. Data are expressed as mean ± SD (*n* = 5). * indicates a significant difference compared with the control group (*p* < 0.05).

As FKC is almost insoluble in water, 1% DMSO was used as a solubilizer and compared with FKCNFs dissolved in water. Figure 8 shows that FKC can be absorbed more efficiently when FKC is prepared as FKCNFs. Without being incorporated within the nanofibers, FKC absorption in all layers does not exceed 5 μg/cm^2^. Figure 8D shows the total dermal absorption (i.e., the total amount of FKC that penetrated into the epidermis and dermis), wherein after 0.5, 2, and 4 h, the total FKC absorbed was 9.42, 18.76, and 19.35 μg/cm^2^, respectively, indicating an increasing trend of FKC absorbed by the skin over time, showing a time-dependent property. The results confirm that preparing FKC into nanofibers can enhance FKC’s percutaneous absorption.

In order to determine the safety of FKCNFs as a cosmetic ingredient, the MoS of FKCNFs was calculated using the test results of percutaneous absorption to evaluate their safe dose in the human body. FKCNFs are intended to be used in face creams, so the value of E_product_ used is 24.14 mg/kg bw/day [35].

From the percutaneous absorption test, the highest dermal absorption was considered, where FKC skin absorption reached 19.35 ± 2.23 μg/cm^2^ after 4 h. The value must be added with the standard deviation and then substituted into the SED formula for calculation. As substances with similar structures mainly have the same or similar metabolic pathways in the human body, it can be inferred that compounds with similar structures may have similar toxicity. Since there is no literature reporting the oral toxicity of FKC, the oral toxicity of FKA with a similar structure was used as a reference, wherein no observed adverse effect level (NOAEL) was observed in mice given 50 mg/kg of orally administered FKA for 25 consecutive days [36]. NOAEL is defined as the highest dose at which no adverse reactions are observed. For cosmetic ingredients, NOAEL mainly comes from 90-day repeated-dose animal studies. As the data from the reference are from a sub-acute study, an adjusted NOAEL was calculated to be 13.89 mg/kg bw/day. This way, the MoS may be computed, giving a value of 190.5. When the MoS value is greater than 100, it means that the component can be used safely. The computation of the MoS for FKCNF is shown in Table 2 below, confirming that the concentration of 1.4% FKCNF is safe to be used as a cosmetic ingredient.

## 3. Materials and Methods

### 3.1. Materials

Flavokawain C (FKC) was obtained from Professor Chih-Hua Tseng (Kaohsiung Medical University, Taiwan); 2-hydroxypropyl-beta-cyclodextrin (HPBCD) from Zibo Qianhui Biological Technology Co., Ltd. (Zibo, China); polyvinylpyrrolidone K120 (PVP), neutral red, and phosphotungstic acid from Sigma-Aldrich (St. Louis, MO, USA); ethanol from Echo Chemical (Miaoli, Taiwan); and B16-F10 cells from Bioresource Collection and Research Center (Hsinchu, Taiwan). Potassium bromide (KBr) was purchased from Scharlab (Barcelona; Spain); potassium dihydrogen phosphate (KH_2_PO_4_) from Ferak (Berlin; Germany); Dulbecco’s Modified Eagle Medium (DMEM) from Himedia Laboratories (Mumbai; India); phosphate-buffered saline (PBS) from Biomate (Kaohsiung; Taiwan); sodium pyruvate, fetal bovine serum (FBS), Pierce^TM^ BCA Protein Assay Kit (BCA), and SuperSignal^TM^ West Femto Maximum Sensitivity Substrate (Femto) from Thermo Fisher Scientific (Waltham, MA, USA); dimethyl sulfoxide (DMSO) and methanol from Aencore Chemical (Surrey Hills, Australia); penicllin–streptomycin–amphotericin B solution (PSA) from Biological Industries (Connecticut, NE, USA); sodium bicarbonate (NaHCO_3_) from Shimakyu Chemical (Osaka, Japan); 3-(4,5-Dimethylthiazol-2-yl)-2,5-diphenyltetrazolium bromide (MTT) from MDBio, Inc. (Taipei, Taiwan); tris (Base), sodium dodecyl sulfate (SDS), glycerol, ammonium persulfate (APS), and dipotassium phosphate (K_2_HPO_4_) from JT Baker (Center Valley, PA, USA); 1,4-Dithio-DL-threitol (DTT) and 99% N,N,N′,N′-Tetramethylethylene-diamine (TEMED) from Alfa Aesar (Lancashire, UK); Coomassie Blue R-250 from Cayman Chemical Company (Ann Arbor, MI, USA); 2-mercaptoethanol from Acros Organics (Geel, Belgium); Immobilon-P PVDF Membrane, RIPA lysis buffer, 50× phosphatase inhibitor cocktail set, and 100× protease inhibitor cocktail set I from Merck Millipore (Burlington, MA, USA); blocking buffer from Visual protein (Taipei, Taiwan); and ascorbyl palmitate (AP) from Propagate Trading Co., Ltd. (Taipei, Taiwan). Primary antibodies: MITF (sc-71588), TRP1 (sc-58438), TRP2 (sc-74439), tyrosinase (sc-20035), ERK 1/2 (sc-135900), p38α/β MAPK (sc-7972), JNK (sc-7345), and GAPDH (sc-32233) from Santa Cruz Biotechnology (Dallas, TX, USA); Phospho-JNK (9225) from Cell Signaling Technology (Danvers, MA, USA); phospho-p38α/β (09-272) and phospho-ERK 1/2 (05-797R) from Merck Millipore (Burlington, MA, USA). Secondary antibodies: anti-Rabbit and anti-Mouse from Merck Millipore (Burlington, MA, USA).

### 3.2. Preparation of FKCNF

Flavokawain C nanofibers were prepared using FES-COS electrospinning equipment (Falco Tech Enterprise Co., Taipei, Taiwan). Briefly, 10 mg of FKC was dissolved in 5 mL of 95% ethanol. Afterwards, 500 mg of HPBCD was added and stirred for 1 h, followed by the addition of various amounts of PVP (50 mg, 100 mg, and 200 mg) with final FKC:HPBCD:PVP ratios of 1:50:5, 1:50:10, and 1:50:20. The prepared nanofiber solution was electrospun at a 0.02 mL/min flow rate and voltage set to 9.6 kV. Afterwards, the dried nanofibers on the aluminum plate collector were gathered and stored in a moisture-proof container. Percent yield was calculated based on the actual FKC content in the FKCNFs using the following formula:

Yield (%) = (actual content/theoretical content) × 100%(1)

### 3.3. FKC Content Analysis by HPLC

FKC content was measured using HPLC-UV (pump model L-2130, autosampler model L-2200, detector model L-2420; Hitachi, Tokyo, Japan). Analysis was conducted using a C18 column (250 × 4.6 mm, 5 µm; Mightysil RP-18) with a mobile phase of methanol/water (9:1) at a flow rate of 1 mL/min. The injection volume used was 20 μL, and detection was carried out at 343 nm. A calibration curve was prepared using FKC at concentrations of 100, 50, 25, 10, 5, 1, 0.5, 0.1, 0.05, and 0.01 μg/mL.

### 3.4. Material Characterization

The surface morphology of the FKCNFs was observed using a Hitachi S4700 SEM (Tokyo, Japan), with the nanofiber diameter calculated using Image J software (National Institutes of Health). In order to determine the appearance of FKCNFs in aqueous solution, 200 μL of 10 μg/mL FKCNF aqueous solution was dropped onto a copper grid for TEM, followed by 200 μL of 0.5% (*w*/*v*) phosphotungstic acid. Once the liquid evaporated, the particle appearance was analyzed by a JEOL JEM-2000EXII transmission electron microscope (Tokyo, Japan). The particle size and polydispersity of FKCNFs were also determined using an Otsuka ELSZ-2000 Zeta-potential & Particle size Analyzer (Osaka, Japan). Crystalline structural changes were evaluated using a Siemens D5000 powder X-ray diffractometer (Munich, Germany) under Cu-Kα radiation (λ = 1.54 Å, 40 kV × 45 mA) over a 2θ range from 2 to 50°, with a scanning rate of 1°2θ/min. IR spectra were obtained using a Perkin-Elmer 200 spectrophotometer (Norwalk, CT, USA) in the range of 4000–1000 cm^−1^ with a resolution of 4 cm^−1^.

### 3.5. Solubility Tests

FKC and FKCNFs were dissolved in hot deionized water with ultrasonication for 10 min, filtered with a 0.45 μm PVDF filter, then quantified using the HPLC-UV method mentioned above.

### 3.6. Stability Tests

Photostability tests were carried out by irradiating FKC and FKCNFs with 266 μW/cm^2^ UVA and 540 μW/cm^2^ UVB energy for 6, 12, 24, 48, and 72 h. Thermal stability tests were carried out by exposure to various temperatures (−20 °C, 4 °C, 25 °C, 50 °C, 65 °C) for 24 h. Afterwards, the FKC content of the samples was quantified using the HPLC-UV method mentioned above.

### 3.7. Cell Viability

To investigate the cytotoxicity of FKCNF, cell viability was assessed by an MTT assay. B16F10 cells were seeded in 96-well plates at a concentration of 1 × 10^4^ cells/mL and incubated for 24 h at 37 °C with 5% CO_2_. The composition of the culture medium was 10% FBS, 1% PSA, 18 mM NaHCO_3_, 1 mM sodium pyruvate, and DMEM (pH 7.2). Afterwards, 100 μL of 20, 10, 5, 1, and 0.5 μM FKC, FKCNF, HPBCD, and PVP was added in sequence.

A primary stock solution was prepared by dissolving 1 mg of the samples in 1 mL DMSO (2 mM stock solution). Each concentration was diluted 10-fold with PBS to 200, 100, 50, 10, and 5 μM, respectively. Finally, the solutions were further diluted 10-fold with DMEM to make the final concentrations of 20, 10, 5, 1, and 0.5 μM. To serve as a control group, DMEM containing 10% PBS was also prepared. After 48 h, the sample was removed and 150 μL of DMEM containing 0.5 mg/mL MTT (3-(4,5-cimethylthiazol-2-yl)-2,5-diphenyl tetrazolium bromide) was added. The solution was removed after 2.5 h, and 100 μL of DMSO was used to solubilize the crystals. The optical density was measured at 550 nm using a continuous-wavelength microplate spectrometer (Spectra Max^®^ ABS plus; Molecular Devices, LLC, San Jose, CA, USA). The cell viability was calculated using the following formula:

Cell viability (%) = (OD_550_ of experimental group/OD_550_ of control group) × 100% (2)

### 3.8. Melanin Content Measurement

B16F10 cells (2 × 10^5^ cells/mL) were seeded in a 6-well plate and incubated for 24 h. After cell attachment, the cells were treated with FKC, FKCNFs, or the positive control ascorbyl palmitate (AP) for an additional 48 h. Afterwards, the wells were washed twice with PBS. Then, the cells were lysed with 200 μL 1% trypsin, and each well was washed with 300 μL PBS. The cell lysates were centrifuged at 12,000 rpm for 10 min, then imaged and analyzed using Image J software. The percentage of melanin content was calculated using the following formula:

Melanin content (%) = (experimental group area/control group area) × 100%(3)

### 3.9. Western Blot Analysis

To investigate the expression of melanogenesis-related proteins in B16F10 cells, a Western blot assay was performed. The B16F10 cells cultured under the same conditions as in the melanin content measurement were lysed using RIPA lysis buffer. The plate was placed on a horizontal shaker for 10 min, then the cells were collected with a plastic scraper. Afterwards, the plate was shaken with an ultrasonic shaker for 10 min and centrifuged at 12,000 rpm for 10 min. The supernatant was taken for protein quantification using a BCA kit. After quantification, 4× sample buffer was added and heated to 100 °C to denature the proteins. The protein sample was separated in a 10% SDS-PAGE electrophoresis gel and transferred to a PVDF membrane at 300 mA for 60 min. The PVDF membrane was placed in a blocking buffer for 1 h, then washed with TTBS 3 times, prior to adding the primary antibodies. It was then incubated at 4 °C in a refrigerator overnight. After washing the membrane with TTBS 3 times, secondary antibodies were added, incubated for 1 h, and then washed with TTBS. The protein targets were detected using enhanced chemiluminescence reagents (Femto) and visualized using Touch Imager (e-BLOT, Shanghai, China). The band expressions were analyzed with Image J software.

### 3.10. Safety Assessment

#### 3.10.1. Percutaneous Absorption

This experiment was conducted with slight modifications to the Guidelines for percutaneous absorption/penetration of the European Cosmetic and Perfumery Association (COLIPA) [37] using Franz diffusion cells. Pig skin was purchased from a local market in Taiwan and confirmed to have no scars, hair, or seals. The subcutaneous fat layer was removed, and the skin was cut into 2 × 2 cm^2^ samples. Prior to conducting the in vitro skin penetration studies, a Delfin VapoMeter^®^ was used to determine the transepidermal water loss (TEWL) of the pig skin to confirm the skin barrier integrity. The TEWL was approximately 6.3 g/m^2^/h. The pig skin was securely sandwiched between the Franz diffusion cell, with the temperature maintained at 32 °C, where the receptor chamber was filled with a buffer solution (0.14 M NaCl + 2 mM K_2_PO_4_ + 0.4 mM KH_2_PO_4_ + 1% PSA) and stirred with a magnet at 600 rpm, while the 500 μg/mL FKC and FKCNF (1.4% FKC) sample solutions were placed in the donor chamber. After 0.5, 2, and 4 h, the pig skins were removed and SC tape stripping was conducted using 3 M adhesive tape to obtain the stratum corneum. The residual skins were heated to 90 °C and separated using a scalpel to obtain the epidermis and dermis. Each layer of the pig skin was soaked in methanol and sonicated for 1 h, then filtered through a 0.45 μm PVDF membrane filter. Afterwards, the FKC content of the samples was quantified using the HPLC-UV method mentioned above.

#### 3.10.2. Calculation for Margin of Safety

According to the “Notes of Guidance for the Testing of Cosmetic Ingredients and Their Safety Evaluation by the SCCS [35]”, the margin of safety (MoS) is calculated based on oral toxicity studies using the following formula:

(4) $$\mathrm{MoS}=\frac{\mathrm{NOAEL}}{\mathrm{SED}}$$

where SED is the systemic exposure dose, and is calculated using the following formula:

(5) $$\mathrm{SED}(\mathrm{mg}/\mathrm{kg}\;\mathrm{bw}/\mathrm{day})=E_{\mathrm{product}}\times \frac{C}{100}\times \frac{{\mathrm{DA}}_{p}}{100}$$

where E_product_ (mg/kg bw/day) is the estimated exposure to a cosmetic product per kilogram of body weight per day, based on the amount and frequency of use, C (%) is the concentration of FKC in the finished product, and DA_p_ (%) is the dermal absorption of FKC under simulated use conditions.

### 3.11. Statistical Analysis

All data are presented as the mean ± standard deviation (SD) of three replicates. The statistical significance of experimental differences was determined by one-way analysis of variance (ANOVA) with Tukey’s post hoc test. A *p*-value ≤ 0.05 indicates statistical significance.

## 4. Conclusions

This study successfully used electrospinning technology to prepare FKC into nanofibers (FKCNFs), improving the physical and chemical properties of the original compound. By improving the particle size, crystal structure, and intermolecular hydrogen bonding, its water solubility and percutaneous absorption were enhanced. B16F10 cell experiments confirmed that FKC and FKCNFs inhibit melanin synthesis, which may be through upregulating the phosphorylation of MAPK family proteins, thereby reducing the expression of the melanin production-related proteins MITF and TRP2, ultimately reducing melanin production. In addition, the MoS of FKCNF calculated using the results gathered in the percutaneous absorption test indicates that FKCNFs can be considered safe for use as a cosmetic ingredient. This study hopes that FKCNFs can be used as a melanogenesis inhibitor in cosmetics in the future to improve dark spots. It is recommended to conduct other in vitro tests, such as skin sensitivity and genotoxicity tests, for more detailed safety assessments for future studies.

## Figures and Tables

**Table 1:** Percent yield and water solubility of FKCNF.

Ratio (FKC:HPBCD:PVP)	Yield (%)	Solubility (μg/mL)
Raw FKC	–	<0.01
1:50:5	85.00 ± 0.80	402.74 ± 13.98
1:50:10	80.19 ± 0.51	436.39 ± 19.95
1:50:20	77.79 ± 0.63	515.32 ± 38.46

**Table 2:** FKCNF margin of safety calculation [35].

Estimated daily exposure (face cream)	E_product_ = 24.14 mg/kg bw/day [35]
Concentration	C = 1.4%
Dermal absorption	DA_p_ = 21.6%
Systemic exposure dose	SED = E_product_ × C × DA_p_ = 0.07
No observed adverse effect level (25-day, daily feeding)	NOAEL = 50 mg/kg bw/day
(90-day, oral)	Adjusted NOAEL = Dose × week × frequency = 13.89 mg/kg bw/day
Margin of safety	MOS = adjusted NOAEL/SED = **190.5**

## Data Availability

The original contributions presented in this study are included in the article/Supplementary Material. Further inquiries can be directed to the corresponding author(s).

## Supplementary Materials

The following supporting information can be downloaded at https://www.mdpi.com/article/10.3390/ijms26072966/s1.
