# Supplementary material for: Physicochemical Characterization, Skin Penetration, Anti-Melanogenesis and Safety Assessment of Flavokawain C Nanofibers
Source: Int J Mol Sci. 2025 Mar 25;26(7):2966. doi: 10.3390/ijms26072966 (PMC11988832; doi:10.3390/ijms26072966)
Supplement: Supplementary file 1 [file ijms-26-02966-s001.zip › ijms-3493321-supplementary.pdf]

Supplementary information for:

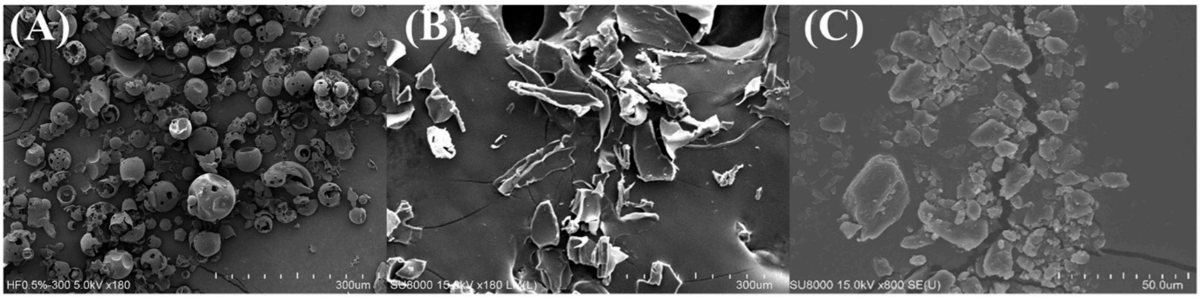

Figure S1. Surface morphology of (A) HPBCD, (B) PVP, and (C) FKC under SEM

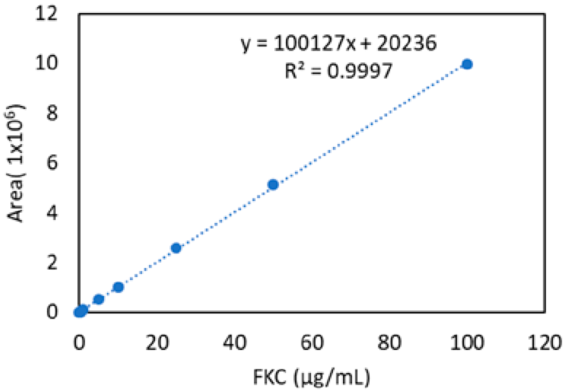

Figure S2. FKC calibration curve (concentration range: 0.01–100 µg/mL).

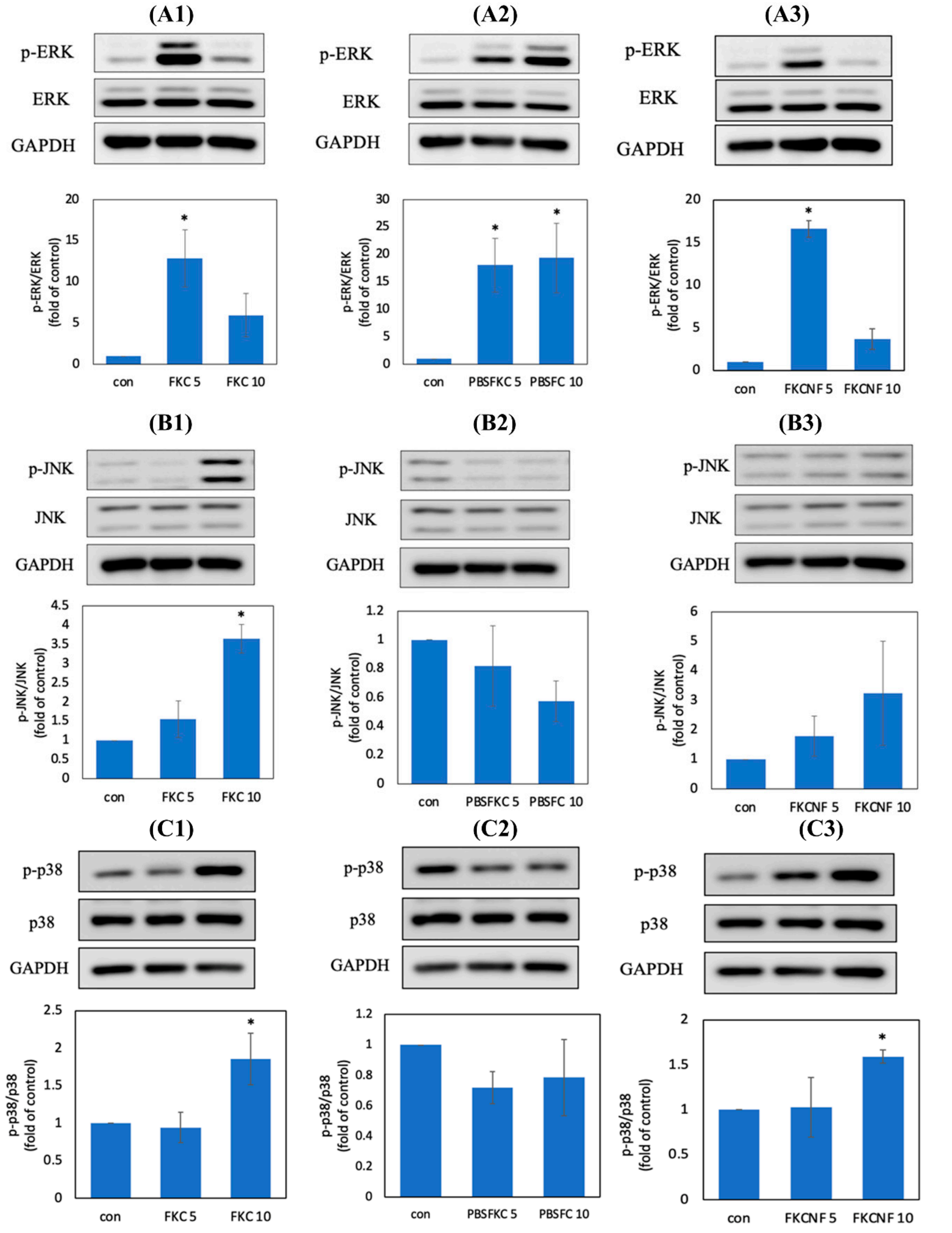

Figure S3. Effect of FKC on the protein expression in the MAPK pathway. Immunoblots and densitometric analysis of (A) p-ERK, (B) p-JNK, and (C) p-p38. The numbers designate the sample groups: (1) 1% DMSO-FKC group, (2) Raw-FKC group, and (3) FKCNF group. GAPDH used as internal control.

* indicates a significant difference compared with the control group ($p < 0.05$)

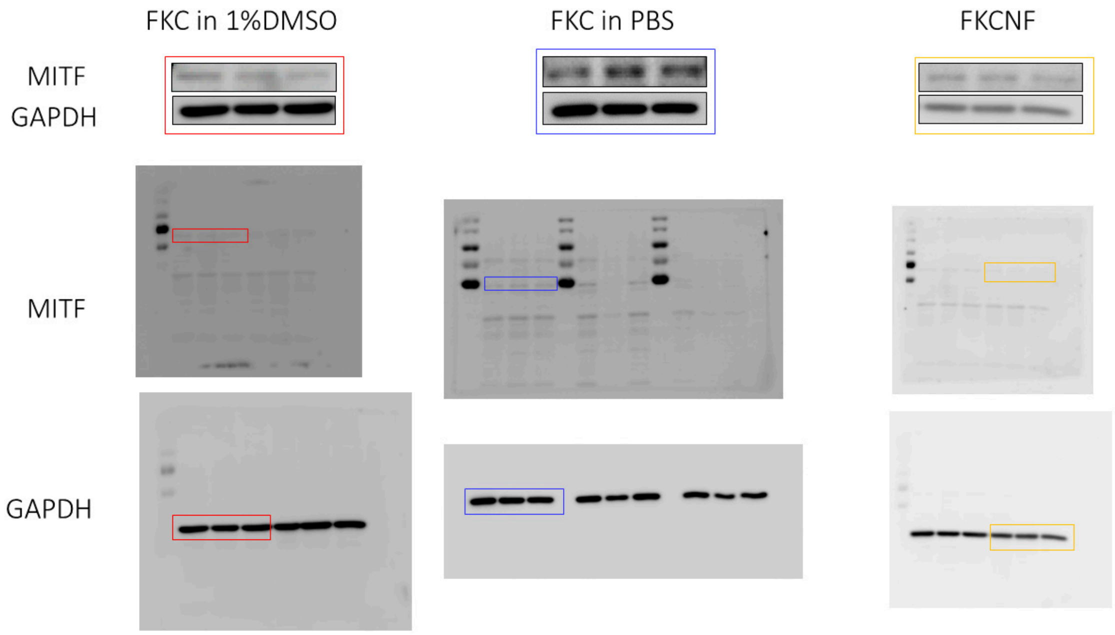

Figure S4. Original unprocessed images of blots for MITF

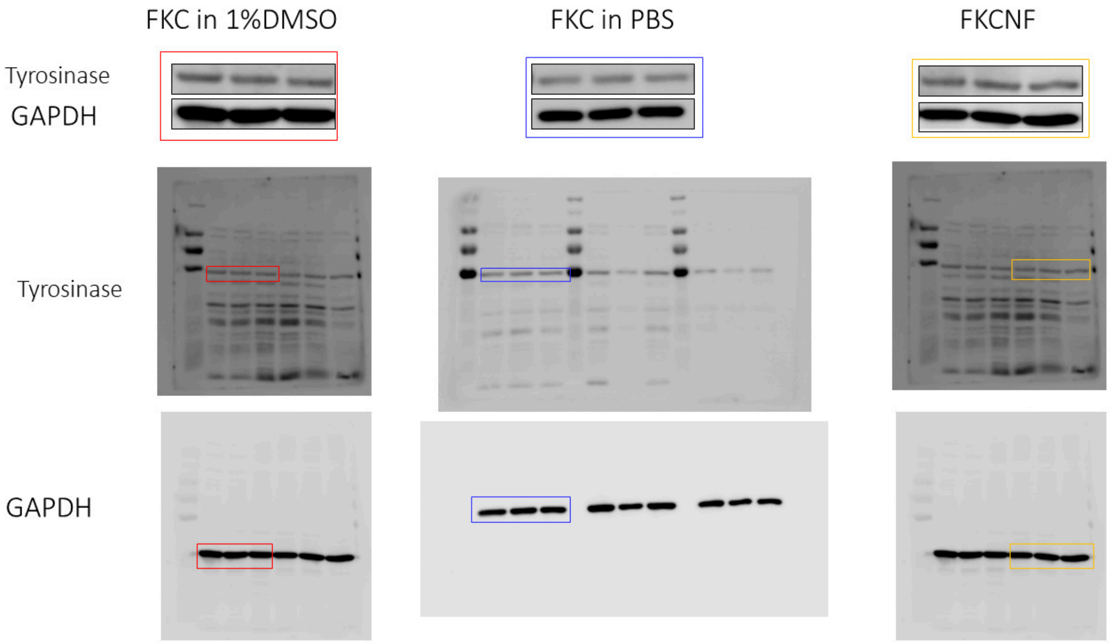

Figure S5. Original unprocessed images of blots for Tyrosinase

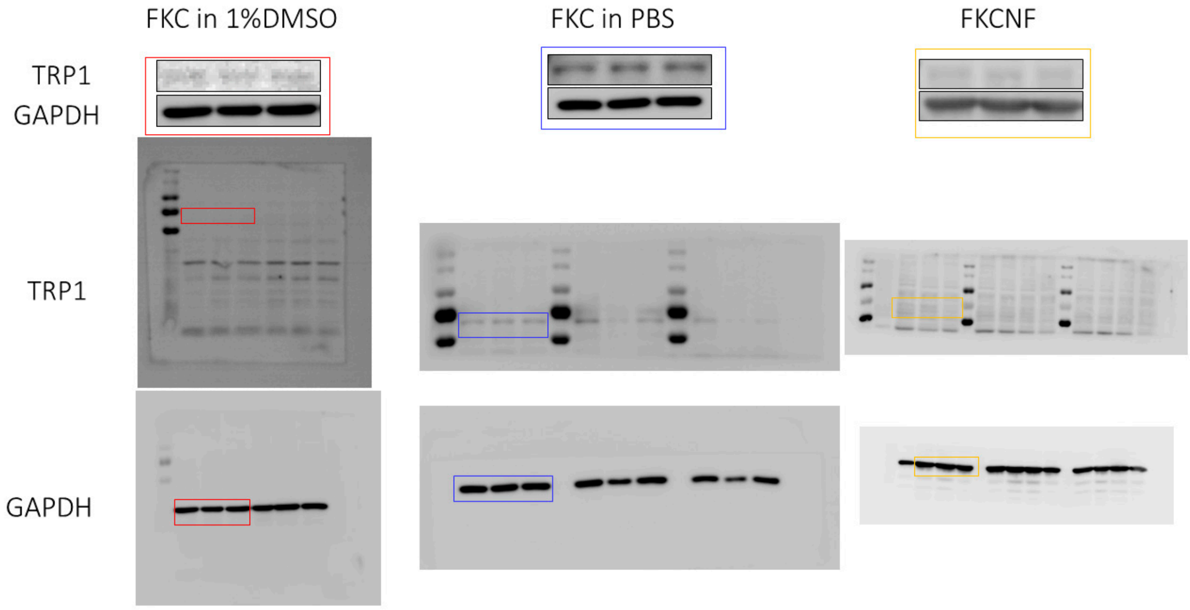

Figure S6. Original unprocessed images of blots for TRP1

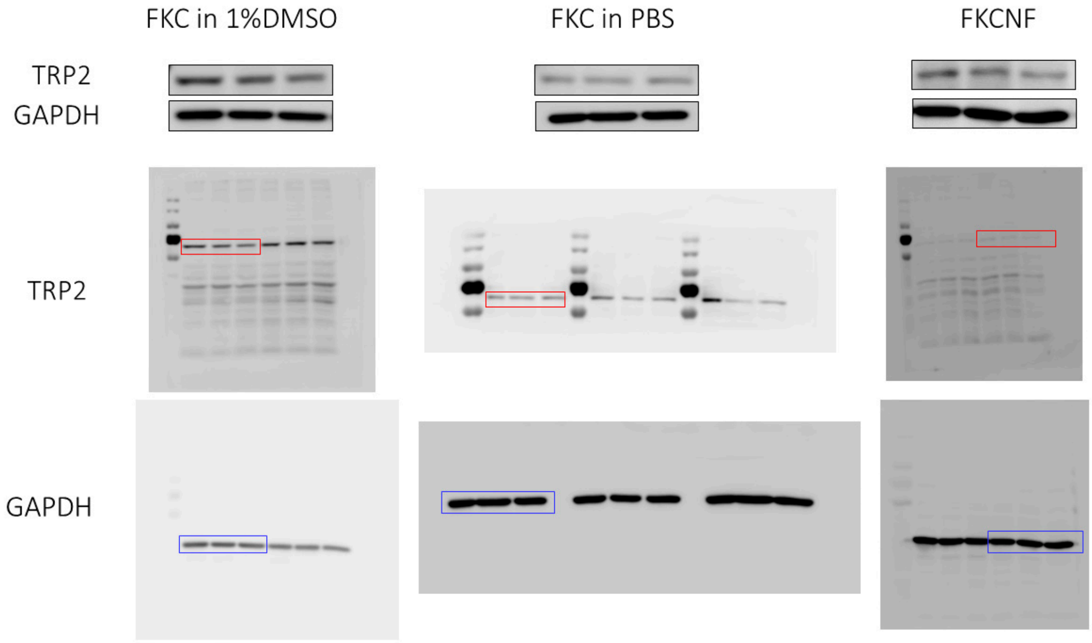

Figure S7. Original unprocessed images of blots for TRP2

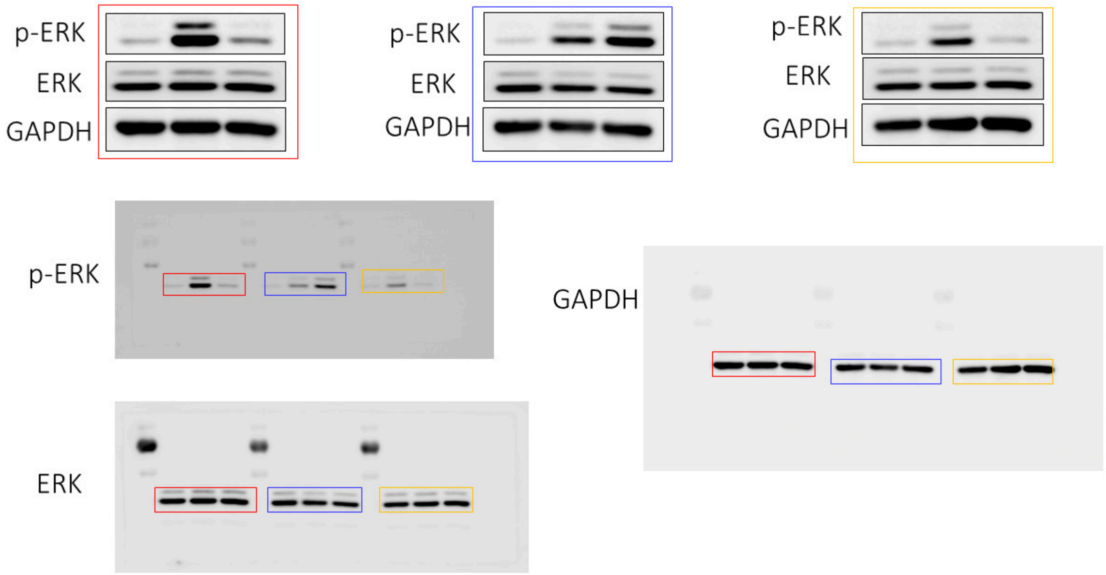

Figure S8. Original unprocessed images of blots for ERK and p-ERK

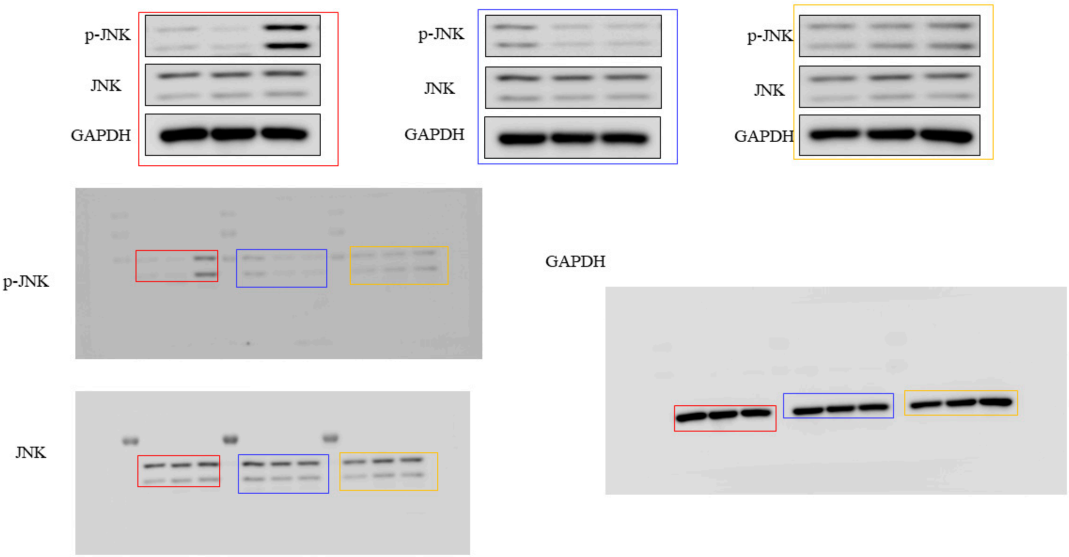

Figure S9. Original unprocessed images of blots for JNK and p-JNK

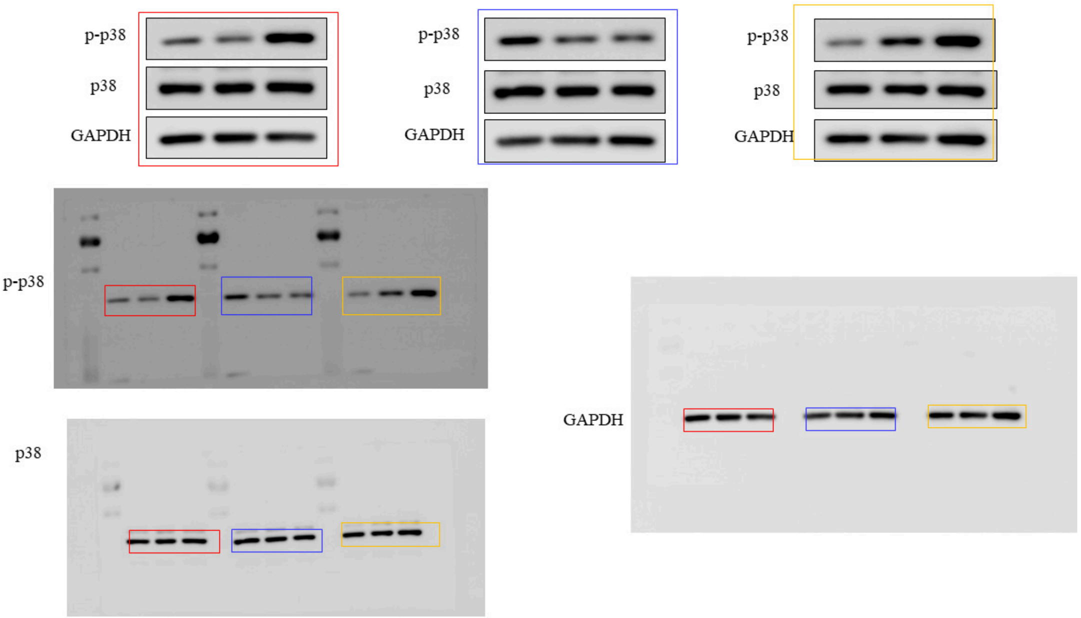

Figure S10. Original unprocessed images of blots for p38 and p-p38
